# Collagen Application in Pediatric Superficial Burns: The Right Time!

**DOI:** 10.1055/s-0043-1777790

**Published:** 2023-12-22

**Authors:** Al-Iqyan Juzar Fidvi, Surendra Kumar Patil, Aniket Jaiprakash Baraskar, Ganesh Deepak Jadhav, Adarsh Kumar Kankam

**Affiliations:** 1 Department of Burns and Plastic Surgery, All India Institute of Medical Sciences (AIIMS), Nagpur, Maharashtra, India; 2 Department of Plastic and Reconstructive Surgery, Government Medical College and Hospital, Nagpur, Maharashtra, India; 3 Department of Surgery, Government Medical College and Hospital Nagpur, Maharashtra, India

**Keywords:** delayed burns, collagen application, healing time, wound infection

## Abstract

**Introduction** Delayed presentation of burn patients, in a developing country with the patient being referred from one center to another higher one, is a common occurrence. Efficient management of such delayed burn wounds thus becomes critical to decrease the morbidity of the patient within economic constraints. The advantages of collagen dressing are numerous. However, there is scarce literature on the timing of its application. Traditionally, it is thought that collagen sheets should be applied within 24 hours of burns as the wound is still sterile. This thus becomes ironical as patients are presenting late. Hence, we studied retrospectively the result of collagen application in delayed presentation of burns.

**Methods** A retrospective study was conducted in which records of pediatric patients of less than 10 years with less than 30% total body surface area scald burns were considered. Collagen dressing was done in all these patients. Presentation time from burns, timing of collagen application, status of wound at various check dresses, complication of burn wound, and total healing times were recorded. Appropriate statistical formulas were used to calculate significance levels for continuous and categorical variables.

**Result** Fifty-three patients, 33 male and 20 female were studied. The most common cause of scald was hot water spillage from baths and cooking, with the anterior trunk being the most involved site. The mean time of presentation of the patient from burns is 71.74 hours and that of collagen application was 76.4 hours. Fourteen (25.4%) patients had wound complications in the form of soakage, fever, and pus. Eight patients had their collagen removed. The average healing time for patients with intact collagen was 12.15 days and that for those on daily dressing was 21.9 days.

**Conclusion** Collagen should be preferred even when the patient presents after 24 hours of burns. A thoroughly washed wound is a necessary prerequisite before collagen application. Burn patients presenting after 3 days have a higher incidence of wound infection. No such time stamp of application of collagen sheets within 24 hours can thus be given for its use as the advantages of adhered and successful collagen dressings outweigh those on daily dressings.

Burn injuries in India are still a potential public health problem, yet these are underrecognized and vastly undermanaged. According to estimates of the National Program for Prevention of Burns Injuries in India, of the 7 million patients who sustain burn injuries each year, 0.7 million require hospitalization, 0.25 million get crippled, and 0.14 million succumb. 1 A common problem that patients face is the timely management of acute burns. As delayed management of the hemodynamic aspect of acute burns is definitely fatal, similarly delay in wound management is crippling and if poorly managed can also be fatal. It is because of the inadequacy of good resources at the grass root level or primary health care centers located in rural parts of India, adequate and efficient management of burn wounds is delayed. This may also stem from the lack of adequate awareness in the population regarding newer treatment methods. Nonetheless, at a tertiary care center in our region, delayed presentation of burn patients in a developing country is a common occurrence and efficient management of such patients is nonetheless expected.

Pediatric Burns is the third most common cause of death in developing countries like India. 2 They are also devastating for the parents both physically and psychologically. Frequent painful dressing changes for wound management can be traumatic for the parent if not economically unviable and risky if done under anesthesia. The advantages and use of biological dressings 3 and dermal substitutes 4 in burns have enumerable references in literature, the details of which are not discussed here. However, the timing of application of collagen from the time of burns is a scantily discussed topic, opinions regarding the application of collagen from the timing of burns are exiguous and also ambiguous. Thus, we retrogradely studied the timing of application of collagen sheet application from the time of burn and factors affecting healing in these burn wounds.

## Materials and Methods

A retrospective study was conducted in the burn unit of our department in which pediatric patients under 10 years of age with scald burns data was collected from patients' records and outpatient follow-up visits. Ethical approval was taken from the ethics committee in our institution. For uniformity only scalds as cause of burn injury and less than 30% total body surface area (TBSA) burns were included in our study. Also, those who presented 24 hours after the burn incident were included. All patients were applied wet meshed collagen available in the hospital supply.

### Method of Collagen Application

**Fig. 1:**
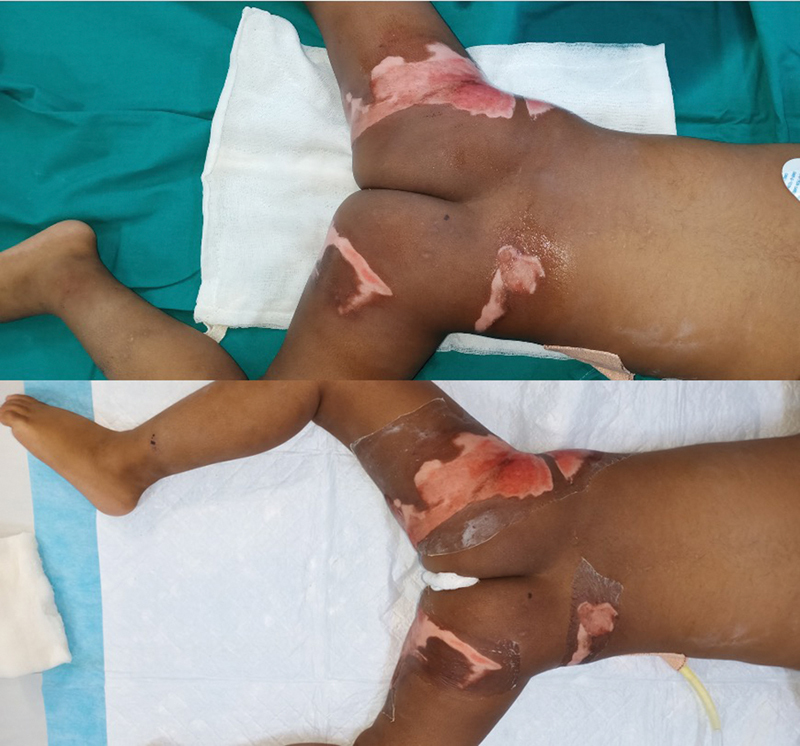
A 2-year-old with perineal and buttock burns. Collagen applied at 76 hours from burns after thorough wash. Note the overlap of collagen on normal skin and transparency after successful drying.

**Fig. 2:**
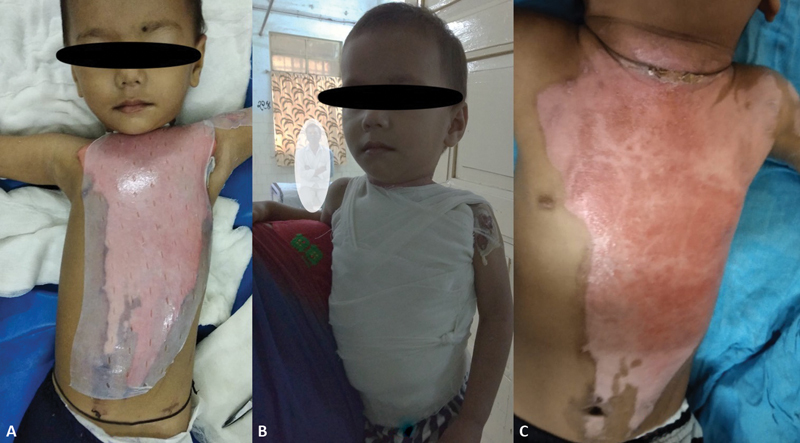
( **A** ) Three-year-old child, collagen applied at 84 hours from burns. ( **B** ) Patient overdressed with Gamgee and comfortable in his mother arms. ( **C** ) At 12 days wound is completely healed.

Once the patient was stabilized and well-resuscitated, they were given triclofos (prodrug: metabolized to trichloroethanol) syrup (500 mg/5 mL) at 50 to 60 mg/kg. 5 After 20 to 30 minutes when the child quietened, the patient was taken to the operating table where the burned skin surface was thoroughly washed with dilute Cetavlon solution, and all dead burnt skin was removed. Blisters were deroofed and burnt skin was removed. The burnt surface was again washed with normal saline and then dried thoroughly. Depending upon the surface area the appropriate size wet meshed collagen (bovine tendon extract, type 1, preserved in isopropyl alcohol for preservative) sheet was used. It was washed thoroughly in normal saline to remove any isopropyl alcohol preservative which may later cause stinging pain to the patient. Then applied over the surface of the burn wound with the back of the forceps evenly spreading it over the wound and removing any bubbles if present ( Fig. 1 ). It was made sure that the collagen covered at least 2 to 3 cm of the normal skin surrounding the burnt skin. This was to make sure that an appropriate seal is provided by the collagen sheet after drying. The applied collagen is dried carefully with a warm air dryer with pendulating hand movements in front of the hair dryer. The endpoint of drying was that the collagen sheet becomes completely transparent and visible wrinkling is evident over the collagen covering the normal skin ( Fig. 1 ). This was followed by covering the burn wound with loose Gamgee rolls (10 cm × 1 m) so as to prevent accidental removal of the sheets with the child's movements and mechanical disturbance and make him comfortable postoperatively ( Fig. 2B ).

**Fig. 3:**
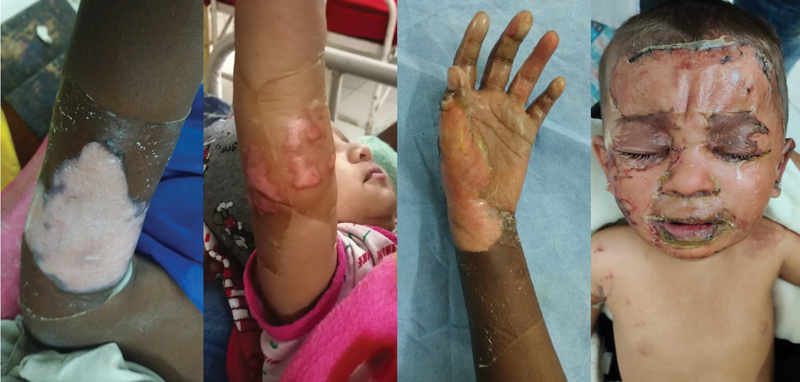
Well-adhered collagen at first check dress in various patients.

**Fig. 4:**
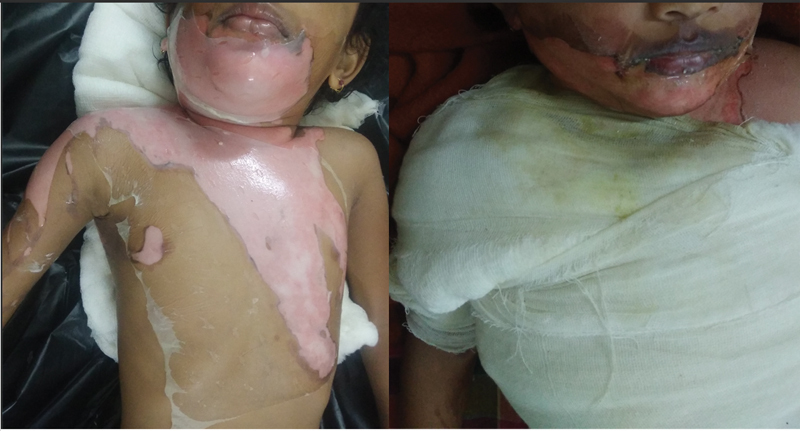
Four-year-old female child, applied collagen at around 90 hours with soakage at day 3. Thus, requiring early check dress.

**Fig. 5:**
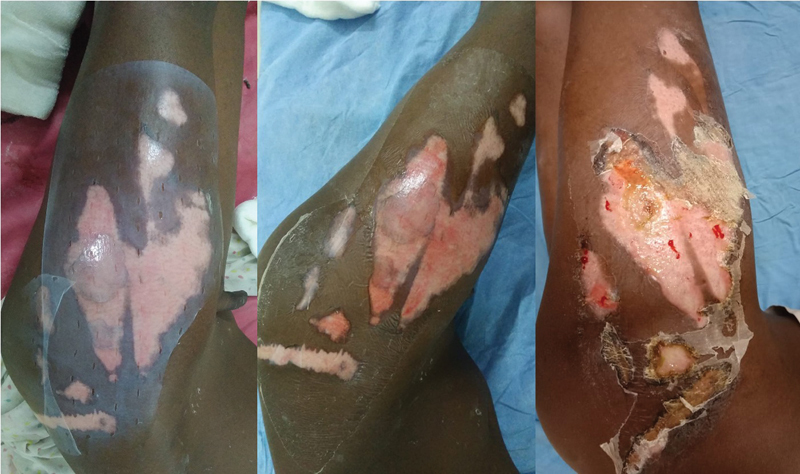
Collagen applied at 60 hours, with good adherence at first check dress and good healing at second check dress.

**Fig. 6:**
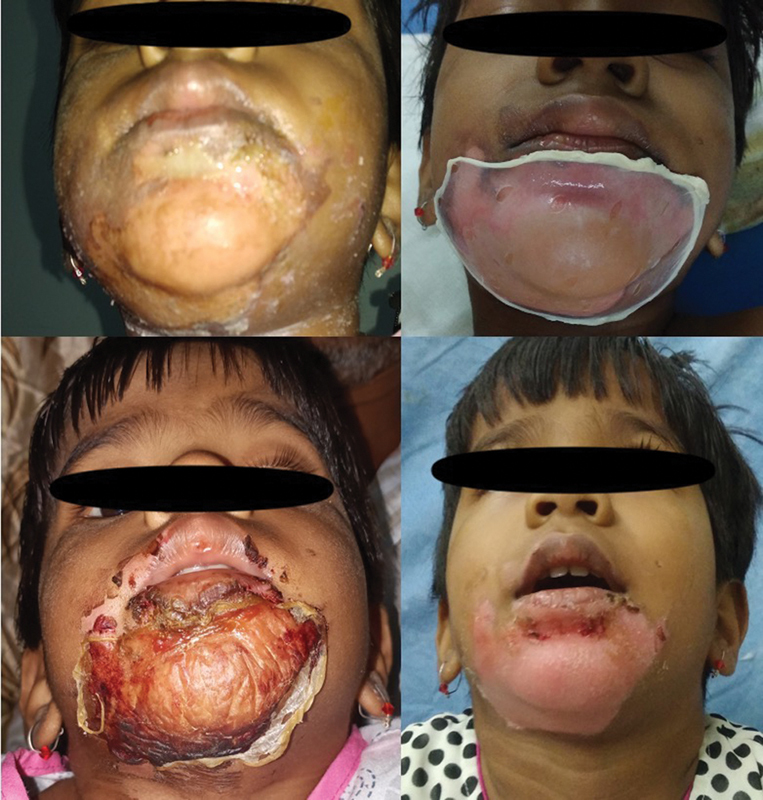
Six-year-old female child presented at 5th day of burns. Collagen applied successfully with good healing at 12 days.

Patient was managed in the ward thereafter. Antibiotics were started as per the antibiotic policy of the hospital. Postoperatively, adequate analgesia and nutrition was maintained. Mobilization was initiated as and when possible. The dressings were done as follows:

(1) At 5th day after collagen application wounds were checked by careful removal of the Gamgee sheet. Any soakage was cleaned, and ointment applied at raw areas (usually meshed sites). The stuck and dry collagen was kept intact. This was the routine first check dress and done irrespective of the status of the wound ( Fig. 3 ).
(2) Irrespective of the day of collagen application if there was any fever or soakage ( Fig. 4 ) visible on the Gamgee dressing the Gamgee was removed. The wound was checked dressed. In case of small pus collection collagen was usually kept intact. Pus was drained from the meshed sites by either puncturing the collagen or removing from meshed site if near, a thorough wash was given, and ointment applied over the meshed regions and again covered with Gamgee dressing. If the amount of pus was extensive and the collagen dressing was not stuck at all, the collagen was completely removed and the patient put on daily dressings.
(3) Second check dress was done around 5th day after first check dress. Depending on the condition of the wound if in the absence of any soakage and dried collagen the wounds were kept open ( Figs. 5 and 6 ). Dressings were changed earlier if there was soakage. If soakage was present the Gamgee dressing was continued. Dressings were also continued in burns around the perineal regions as these were prone to frequent soakage. Depending on the status of the wound thereafter necessary dressings were done or the dried adhered collagen was kept open.

The collagen was trimmed as and when the wound healed usually from the periphery. The percentage of area healed was noted on each check dress.

All data regarding demography of patient, date and type of burn, presentation of patient to our center, timing of collagen application, post-collagen application progress of the patient including signs and symptoms of infection, number of dressing, and time of healing of the wound was extracted from patient records.

Data were entered in MS Excel and analysis was done using SPSS 21.0 version. Data were presented as mean and standard deviation for continuous variable and as percentages for categorical variables. Unpaired *t* -test was done to compare two-group means and analysis of variance for more than two-group means. Pearson's correlation was done to correlate two continuous variables. Chi-square test was done to find out association between categorical variables. A *p* -value of less than 0.05 was considered significant.

## Results

Of the 53 patients ( Table 1 ) included in the study, there were 33 male and 20 female children. The most common cause of burns in these children was from hot water spillage (56.6%) followed by hot curry and tea spillage. The mean area of TBSA burns was around 15%. Maximum number of cases were between 11 and 20% ( *n* = 30, 56.5%) followed by those ≤ 10% TBSA ( *n* = 14, 26.4%) ( Table 2 ).

**Table 1:** Patient demography

Male	Female	Total	
33	20	53	
Mean age	4.5 y		
Mean TBSA involvement	15.21% TBSA		
Cause of hot burns ( *n* = 53)			
Hot water	30 (56.6%)		
Hot curry	11 (20.8%)		
Tea	9 (17.0%)		
Soup	2 (3.8%)		
Hot oil	1 (1.9%)		
Site of burns ( *n* = 53)		***n***	**%**
Head and neck		2	3.8
Anterior trunk		31	58.5
Posterior trunk		6	11.3
Upper limb	Arms	9	17.0
	Forearms	7	13.2
	Hand	5	9.4
Buttocks		5	9.4
Lower limbs	Thighs	22	41.5
	Legs	13	24.5

Abbreviation: TBSA, total body surface area.

**Table 2:** Total healing time and percentage of burns

%TBSA burns	*N*	Total healing time from day of burns		ANOVA test *p* -value
		Mean	SD	
≤ 10%	14	11.2143	1.67233	0.017
11–20%	30	14.3333	3.76310	
21–30%	9	17.0000	8.95824	
Total	53	13.9623	4.96512	

Abbreviations: ANOVA, analysis of variance; SD, standard deviation; TBSA, total body surface area.

The anterior trunk was the most commonly involved region (58.5%). This was followed by thighs (41.5%) and legs (14%). The involvement of arm, forearm, and hands was 17, 13, and 9%, respectively. The least involved regions were the head and neck, posterior trunk, and buttocks to the tune of 4, 11, and 9%, respectively ( Table 1 ). This was considering the fact the there were multiple burn areas in a single patient.

At the time of presentation 70% of the children had open wounds which were not dressed and had dried exudate with necrotic skin. Of these two patients had Colgate (toothpaste) and one patient had lime whitewash applied over the wounds. The remaining 30% patients had closed dressed wounds. Five of these patients did have closed dressing but they were soaked with foul smell.

The mean time of presentation of the patient from the time of burns was approximately 71 hours ( Table 3 ). Collagen was applied on average within 4.6 hours of patient presentation in the hospital. The earliest that was applied was immediate (within 1 hour) on presentation and the most delayed was 16 hours after presentation. Note that 54.8% ( *n* = 29) had their collagen applied between 48 and 96 hours of presentation and stabilization, 22.6% cases between 96 and 120 hours, and another 22.6% within 48 hours. The time of collagen application was on an average 76.5 hours from the time of burns.

**Table 3:** Timing of presentation and collagen application

	Mean	SD	Minimum	Maximum
Time of presentation from burn injury (h)	71.74	26.82	26	116
Time of collagen application (h)	76.40	26.94	30	120
Time difference between presentation and collagen application (h)	4.66	2.59	1	16
**Time of collagen application**	***N***		**Percentage**	
≤ 48 h	12		22.6	
48–72 h	13		24.5	
72–96 h	16		30.2	
96–120 h	12		22.6	
Total	53		100.0	

Abbreviation: SD, standard deviation.

Of the 53 patients, 39 patients (73.6%) had their first check dress done on day 5 ( Table 4 ). They had no signs and symptoms of wound infection in the form fever, soakage, or pus drainage from wound site. The collagen was intact in these cases and was thus redressed with fresh Gamgee rolls.

**Table 4:** First check dress

First check dress (d)	Frequency	Percentage
2	5	9.4
3	5	9.4
4	4	7.5
5	39	73.6
Total	53	100.0

**Fig. 7:**
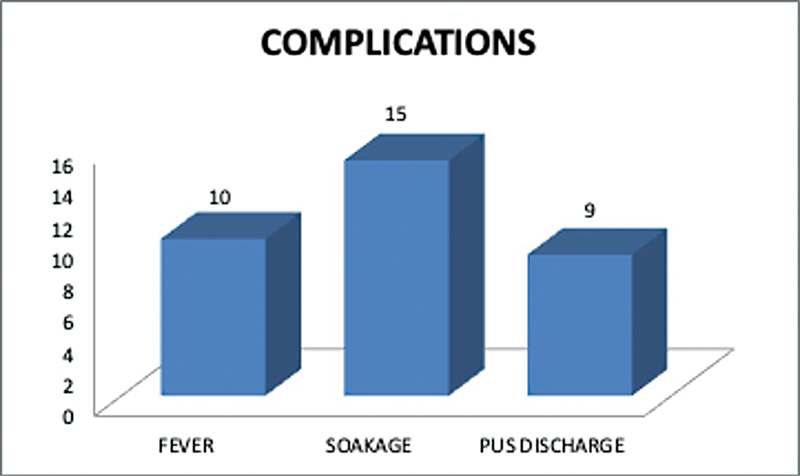
Incidence of fever, soakage, and pus at wound site.

Of the remaining 14 (25.4%) patients, all 14 (28.3%) had wound soakage, 10 (19%) patients had fever, and 9 (17%) had pus discharges after collagen application ( Fig. 7 ). These symptoms resulted in these patients having their first check dress earlier, with five (10%) of them at day 2, five (10%) on day 3, and four (7%) on day 4.

The collagen was intact completely ( Table 5 ) over the TBSA of burns in 41 (77.4%) cases. The collagen was totally removed in 8 cases (15.1%). All these cases had pus in their wounds with completely loosened collagen which could not be managed with local drainage from the meshed site. The remaining four cases (7.6%) had partially intact collagen at some sites and unstuck collagen at other sites. The intact collagen was covered with fresh Gamgee and the sites from where the collagen was removed were put on daily dressing. Daily dressings were thus done for 12 patients including those who had collagen completely removed ( *n* = 8) and in whom it was partially removed ( *n* = 4).

**Table 5:** Status of collagen at first check dress

State		*N*	Percentage
Completely intact		41	77.4
Collagen removed completely		8	15.1
Partially intact	Chest collagen intact, bilateral thigh collagen removed	1	1.9
	Face and lower limb intact, upper limb collagen removed	1	1.9
	Trunk collagen intact, thigh collagen removed	2	3.8
Total		53	100.0

At second check dress which was usually done at the 5th day after the first check dress, 26 patients (49%) had dried dressing and intact collagen and these were kept open thereafter.

Nineteen cases (36%) had some soakage present. These had intact collagen over the wound with small serosanguinous collections at multiple pockets which were removed through meshes. Dressing was continued in these patients. The third check dress in these patients showed no soakage and were kept open thereafter. The remaining eight patients (15%) were on daily dressing as there collagens were removed.

There was a significant correlation ( Table 6 ) when duration of presentation of patient was correlated with presence of wound infection. All the 14 patients who had signs and symptoms of wound infection and required early check dress had presented after 85 hours.

**Table 6:** Correlation between time of presentation and presence of wound infection

Variables		Time of presentation (h)			Time of collagen application (h)		
		Mean	SD	*p* -Value	Mean	SD	*p* -Value
Fever	Present	88.20	15.98	0.030	94.00	16.17	0.020
	Absent	67.91	27.50		72.30	27.42	
Soakage	Present	85.73	21.68	0.015	91.40	21.51	0.009
	Absent	66.21	26.87		70.47	26.79	
Pus discharge	Present	94.00	13.27	0.005	99.56	13.22	0.004
	Absent	67.18	26.69		71.66	26.64	

Abbreviation: SD, standard deviation.

The mean healing time from the day of burns was 13.96 days for the 53 patients. However, in the patients in whom the collagen was completely intact ( *n* = 41, 77.4%) and partially intact ( *n* = 4, 7.5%), the mean healing time in these patients was 12.25 days ( Table 7 ).

**Table 7:** Average healing time in intact collagen patients and those in whom collagen was removed and were on daily dressings

**Time of collagen application**	**No. of cases**	**Collagen intact**	**Partially intact**	**Average healing time (d)**
≤ 48 h	12	12	0	12
48–72 h	13	12	1	12.7
72–96 h	16	9	3	11
96–120 h	12	8	0	12.75
Total	53	41	4	12.15
Collagen intact in patients		45		
**Time of collagen application**	**No. of cases**	**Collagen removed completely**	**Collagen partially removed**	**Average healing time (d)**
≤ 48 h	12	0	0	
48–72 h	13	0	1	20
72–96 h	16	4	3	21.5
96–120 h	12	4	0	24.5
Total	53	8	4	21.9
Collagen removed with patient on daily dressings		12		

On the other side, in the patients in whom collagen was removed completely ( *n* = 8, 15%) and partially removed ( *n* = 4, 7.5%) and were on daily dressing the average healing time was 21.9 days ( Table 7 ). Of these two patients healed at 30 and 35 days, both required skin grafting. Thus, when total healing time in patients was correlated with the intact collagen dressing ( Table 8 ), there was significant reduction in healing times as compared with those in whom collagen was removed and were on daily dressings ( *p* 0.001).

**Table 8:** Time of healing with status of collagen dressing

Collagen	Total healing time from day of burns		ANOVA test *p* -value
	Mean	SD	
Intact	12.15	1.61	< 0.001
Partially removed	12.505	0.58	
Completely removed	24.005	5.71	
Total	13.965	4.97	

Abbreviations: ANOVA, analysis of variance; SD, standard deviation.

**Fig. 8:**
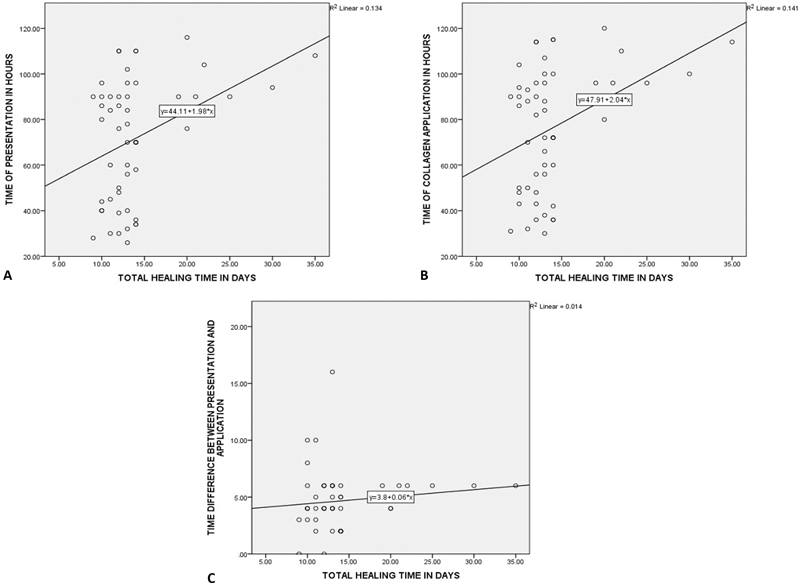
Pearson's correlation chart showing relation with time of presentation, collagen application, and healing.

When total healing times were correlated with the time of presentation of burns and with the time of collagen application, there was a significant correlation between the two ( Table 9 ) ( Fig. 8A and B ). However, the difference in timing of collagen application from the time of presentation of the patient was not significant when correlated with healing of wounds ( Table 9 ) ( Fig. 8C ). The average time in which collagen was applied to a patient after he or she reported to us was 4.6 hours. As seen in Fig. 8C , this duration was constant in maximum number of cases and thus did not affect healing times. The collagen should be applied as early as possible from presentation.

**Table 9:** Total healing time and presentation of patient and collagen application (also see Table 2)

	Pearson's correlation	*p* -Value
Total healing time vs. time of presentation in hours	0.366	0.007
Total healing time vs. time of collagen application in hours	0.376	0.006
Total healing time vs. time difference between presentation and application	0.119	0.397

As mentioned earlier ( Table 6 ), though it was observed that late presentation was associated with wound infection and subsequent loss of collagen dressing, however, collagen dressing was still intact in 17 (32.1%) cases who presented after 72 hours and healed on an average in 11.9 days ( Table 7 ). Thus, healing time was not affected when collagen dressing was successfully applied even when the patient presented late.

Another significant factor which affected healing time was percentage of burns ( Table 2 ). The more the percentage of burns, more incidence of wound sepsis was seen which affected healing times. Of the nine patients with 21 to 30% burns, two had their collagens removed completely and healed on 30th and 35th day. One patient had partially removed collagen which healed at around 25th day. In the remaining patients, even though collagen was intact, persistent soakage was seen in their wound.

## Discussion

In concordance to the world report by the World Health Organization on Child Injury and Prevention, 6 we also reported trunk to be the most common site of burn injuries from scald. And the most common reason reported was from hot water spillage.

The concept of the usage of biosynthetic material for burn wound materialized from the observation that wounds left with blister intact healed with minimal necrosis. 7 However, according to the same study biosynthetic materials are best applied to early, moist surfaces, before desiccation and surface necrosis and not more than 2 to 6 hours post-burn. The effect of biosynthetic substitutes mainly depend on their ability to change the fate of the zone of stasis in a favorable manner. 7 8 Following this discovery enumerable biosynthetic materials have been made available among which the collagen has been more commonly used as it is the cheapest and easily available in our country. Collagen sheets when applied on burn wound surface provide an impermeable physiological interface with the environment. By inhibiting metalloproteinases, promoting angiogenesis, and providing an impermeable scaffold, collagen sheets serve as a template for infiltration of macrophages and fibroblast. Eventually, the collagen deposited by the fibroblasts replaces the collagen of the sheet. 9 10

It has been exhaustively discussed by Young and Grinnell 11 and Neely et al 12 that metalloproteinase activity especially which comes into play at day 2 and peaks at day 4 from the day of burns prevented burn wound epithelization and favored granulation formation. Collagen thus by preventing this aggravated metalloproteinase activity favors early wound epithelization and wound healing.

Traditionally, we have been taught that collagen dressing was futile after 24 hours of burn injury in view of colonization of the wound surface. Though there is lack of evidence to support this advice, it can be speculated so because of possible bacterial colonization and adhered biofilm formation over the burn wound surface. Three studies from developing countries 13 14 15 have associated delayed presentation of burns with higher rates of wound infection and sepsis and ultimately survival. An admission delay by 78 hours has been critically associated with higher rates of wound infection in moderate burn patients. A similar study by Sheridan et al 16 in children has also associated delay in presentation (> 24 hours) from day of burns with higher incidence of wound sepsis, bacteremia, and renal dysfunction. However, the TBSA of burns in these children was more than 50%. In our study, the TBSA was kept below 30% as an inclusion criterion for the study even then we found a significant difference in wound infection rates and healing time with > 20% TBSA burns having more wound infections, collagen removal, and longer healing times (17 days) as compared with those with < 20% TBSA burns (13 days). Thus, it can be inferred that delayed presentation of patient with greater body surface area of burns has significant impact on wound sepsis.

Bacterial colonization of the burn wound occurs after 24 hours, till then the burn wound surface is sterile. Gram-positive bacterial colonization occurs first, then followed by Gram-negative organisms. Most of the organisms which colonize the burn wound are biofilm forming. 17 These include *Staphylococcus aureus* , *Pseudomonas aeruginosa* , and Acinetobacter species. This glycocalyx containing biofilm which forms after 48 hours can be a significant factor that may prevent collagen from sticking. The biofilm when formed is reversible and immature and easily washed from the surface. Thus, thorough washing with any cleansing agent can successfully remove the biofilm and with it the bacterial colonies. The wounds can thus become sterile and favorable for collagen application especially in cases who present late after burn injury. Also, meshing of the collagen provides a drainage for any collection which prevents removal of the collagen.

Numerous previous studies have successfully used collagen over burn wounds, but the timing of collagen application is insufficiently discussed. In 2001, in a case series by Delatte et al 18 225 pediatric burn cases were reviewed in which only new clean cases not involving critical areas were included. Beta glucan collagen was applied in 43 cases. Several days old (exact number not mentioned) wounds which were considered contaminated were deemed unsuitable for collagen application. They noted that the outcome of burn injuries when collagen dressing was compared with daily silver sulfadiazine dressing was similar except the fact that daily wound care and painful dressing was avoided in the β-glucan collagen group. In contrast, in our series of 53 cases in which all patients had collagen dressing the healing time was 12 days as compared with 22.5 days when patients were put on daily dressing irrespective of the time of presentation of patient.

In a study by Tayade et al, 19 a comparison was made between 25 cases in whom collagen was applied with silver sulfadiazine dressings. Only burns with < 15% TBSA and presenting within 24 hours (1 day) were included in the group. Patients presenting more than 24 hours were excluded. Similarly, a study by Rai et al in 2013 20 also included patients presenting within 48 hours of burns for collagen application. No reason has been cited for not applying collagen in delayed presentation. Both of these studies showed decreased healing times with collagen dressing as also observed in our series. However, in our study similar healing times were also observed with cases presenting late and with delayed collagen application up to 5 days from burns. Contrast to our study, Khurram et al 21 and Kumar et al 22 included cases who presented within 24 hours only. No explanation has been discussed as to why such inclusion was taken. However, the mean healing times in both the studies were 11 and 13 days, respectively. We also achieved similar healing time period of 12.5 days in our cases in whom collagen was intact and healed without complications.

In a study similar to ours by Waghmare et al 23 100 pediatric burn cases were considered in whom collagen dressing was applied to everyone. Only 20 cases presented after 24 hours. They showed that good healing was achieved in their 92 cases with average healing time of 7 to 10 days. Five of their cases who had delayed presentation developed wound site infection necessitating collagen removal. Thus, they showed good healing can be achieved with delayed collagen application. Similarly, in our study where all collagen application was delayed 45 cases (84%) cases healed and 8 cases (15%) required collagen removal.

Consistent with our findings, Ramakrishnan et al 24 have shown increased incidence of infection at wound site after 72 hours of patient presentation. In our series cases presenting after 85 hours had increased incidence of wound sepsis. Thus, a 3-day period can be proposed to have increased incidence of burn wound infection from the time of burns though subject to many other factors.

Percentage of burns is a major factor which affects wound status. The greater the percentage of burns the more the chances of patient developing wound sepsis. Numerous references have shown an association between burn surface and complications in burn patients including wound sepsis. 14 16 25 To avoid any bias, we included only < 30% TBSA burns in our series. Nonetheless, we also reported higher wound infection and thus delayed healing ( Table 2 ) in patients with increasing burn surface area.

## Conclusion

Timely intervention and efficient management of burn wound is the key to an uncomplicated early healing. Though subject to other factors, a 3-day delay in presentation after burns can be considered to have higher chances of developing wound infection and sepsis. Collagen dressing with all its advantages should be the mainstay in dressing of burn wounds especially superficial wounds in children. There should not be any doubt whether to not apply collagen over burn wounds because if successfully applied and if the collagen is well adhered healing of wound is definitely fastened. From our study we can conclude, as such no time stamp can be put on the timing of collagen application. Further, it is advised that even if the presentation of the burn patient is delayed, collagen dressings should be encouraged over thoroughly cleaned wounds as it definitely improves outcomes in terms of earlier wound healing, lesser dressings, and anesthetic risks. Appearance of granulation on wound may be considered as a contraindication.
